# Fuzzy Lexical Representations in Adult Second Language Speakers

**DOI:** 10.3389/fpsyg.2021.732030

**Published:** 2021-11-19

**Authors:** Kira Gor, Svetlana Cook, Denisa Bordag, Anna Chrabaszcz, Andreas Opitz

**Affiliations:** ^1^Graduate Program in Second Language Acquisition, School of Languages, Literatures, and Cultures, University of Maryland, College Park, MD, United States; ^2^National Foreign Language Center, University of Maryland, College Park, MD, United States; ^3^Herder Institute, University of Leipzig, Leipzig, Germany; ^4^University of Haifa, Haifa, Israel; ^5^Department of Psychology, University of Pittsburgh, Pittsburgh, PA, United States; ^6^Center for Language and Brain, HSE University, Moscow, Russia

**Keywords:** L2, L1, fuzzy, lexical representation, word recognition, lexicon, word learning

## Abstract

We propose the fuzzy lexical representations (FLRs) hypothesis that regards fuzziness as a core property of nonnative (L2) lexical representations (LRs). Fuzziness refers to imprecise encoding at different levels of LRs and interacts with input frequency during lexical processing and learning in adult L2 speakers. The FLR hypothesis primarily focuses on the encoding of spoken L2 words. We discuss the causes of fuzzy encoding of phonological form and meaning as well as fuzzy form-meaning mappings and the consequences of fuzzy encoding for word storage and retrieval. A central factor contributing to the fuzziness of L2 LRs is the fact that the L2 lexicon is acquired when the L1 lexicon is already in place. There are two immediate consequences of such sequential learning. First, L2 phonological categorization difficulties lead to fuzzy phonological form encoding. Second, the acquisition of L2 word forms subsequently to their meanings, which had already been acquired together with the L1 word forms, leads to weak L2 form-meaning mappings. The FLR hypothesis accounts for a range of phenomena observed in L2 lexical processing, including lexical confusions, slow lexical access, retrieval of incorrect lexical entries, weak lexical competition, reliance on sublexical rather than lexical heuristics in word recognition, the precedence of word form over meaning, and the prominence of detailed, even if imprecisely encoded, information about LRs in episodic memory. The main claim of the FLR hypothesis – that the quality of lexical encoding is a product of a complex interplay between fuzziness and input frequency – can contribute to increasing the efficiency of the existing models of LRs and lexical access.

## Introduction

This article introduces the fuzzy lexical representations (FLRs) hypothesis with a focus on adult second language (L2) learners. It outlines the construct of the FLR that is characterized by imprecise, or fuzzy encoding of its form and/or meaning, and potentially, the mapping between them. Less distinct boundaries of FLRs result in their reduced differentiation from neighboring representations in the mental lexicon. Fuzziness is primarily a property of less familiar words that occur less frequently in the input, both in the native language (L1) and L2; however, two factors contribute to its much greater pervasiveness in L2 than L1. First, less familiar words are more numerous in the L2 mental lexicon than the L1 mental lexicon. And second, for the reasons discussed below, L2 speakers experience more difficulties with encoding the phonological form and meaning of L2 words, establishing strong mappings between them, and integrating new L2 lexical entries in the mental lexicon compared to L1 speakers. In particular, more LRs in L2 retain fuzzy phonological encoding even for more familiar words.

Accordingly, we will refer to the empirical evidence primarily on L2, but also on L1 lexical processing, as appropriate. While a lexical representation (LR) in literate L2 speakers^1^ encodes both the sound and the written form of the word, we will treat auditory encoding as the core aspect of form encoding. Indeed, the spoken modality is the primary source of input for a majority of monolingual speakers and also bilinguals/monolinguals, who learn languages beyond a traditional foreign language classroom with a strong emphasis on written input. We believe that, in addition to being more ecologically valid, this approach helps to better address the L2-specific sources of fuzziness and to offer suggestions for the development of testable models of auditory L2 word recognition. The focus on auditory LRs makes it possible to rely on the existing literature on the topic when more research on the role of phonological encoding than orthographic encoding in FLRs is available.

In the following sections, we will discuss the properties of FLRs in L2 and explore the dynamics of their engagement in the process of word recognition. In particular, we will address the following questions associated with fuzziness in L2 LRs:

- Why is there a need for a FLR construct?
- Is the construct of FLR new, or does it rename existing constructs?
- What causes the fuzziness of L2 LRs?
- What consequences does fuzziness in LRs have for L2 word recognition and lexical processing?
- How do FLRs develop over time – is fuzziness reduced?
- How can the construct of FLRs contribute to increasing the efficiency of the existing models of LRs and lexical access?

The FLR hypothesis is based on the idea that imprecise and ambiguous linguistic encoding of any component of the LR (phonological, orthographic, and lexical-semantic)^2^ has important consequences for several aspects of L2 word storage and retrieval. First, poor encoding at one or more levels leads to weak form-meaning connections and, sometimes, incorrect form-meaning mappings. L2 speakers tend to confuse L2 words that are less similar-sounding more than L1 speakers do, that is, there is a greater Levenshtein distance between confusable L2 words than confusable L1 words (see Levenshtein, 1966; Cook et al., 2016). Second, poor phonological encoding leads to a low level of lexical activation and competition of individual LRs in spoken word recognition, because FLRs are not clearly separated from their phonological neighbors and none of the competing LRs is treated as a clear “target.” At the same time, a larger set of word candidates including phonologically more distant words, which would not normally be activated in L1, gets activated, albeit at lower activation levels. And third, fuzzy phonological encoding of LRs may persist in L2 and interact with input frequency in such a way that the quality of encoding may not necessarily improve with repeated encounters with the word in the input. Accordingly, input frequency, while being an important factor in shaping LRs, is not the sole determining factor in resolving fuzziness of LRs in L2. Together, the unfaithful encoding and the mapping problems produce nonnative patterns of lexical activation, competition, and selection in L2 word recognition.

The FLR hypothesis accounts for a range of phenomena observed in L2 lexical processing, including well-documented lexical confusions in comprehension and production, slow lexical access, retrieval of incorrect lexical entries, weak lexical competition, reliance on sublexical rather than lexical heuristics in word recognition, the precedence of word form over meaning, and the prominence of detailed, even if imprecisely encoded, information about LRs in episodic memory.

## Why Is There a Need For a Flr Construct?

A number of phenomena observed in L2 word processing and learning seem to be associated with one core property characterizing nonnative LRs. According to the FLR hypothesis, this property is unfaithful or fuzzy encoding. The FLR hypothesis seeks a principled account of fuzziness characterizing L2 LRs and explores the sources of fuzziness in L2 lexical processing.

L2 speakers are notorious for confounding words, with numerous examples of misunderstandings observed in L2 oral and written productions (Dušková, 1969; Hemchua and Schmitt, 2006). They are unsure whether and how *truck* is different from *trunk*, *helmet* from *hamlet*, *lie* from *lay*, or *accident* from *incident* (a form-related confusion). At beginning stages of L2 acquisition, L2 learners may experience uncertainty about whether *trunk* means “suitcase,” a “tree part,” or both, and how these meanings can help to make an informed guess about what the elephant’s *trunk* is (a meaning-related confusion). Both meaning- and form-related aspects of the development of robust lexical representations in L2 are prone to difficulties.

When learning new vocabulary, L2 speakers struggle to establish a strong connection between the semantic representation for a novel word with its form, especially when inferring the meaning from sentence context or extended context, in which the word occurs (see Bordag et al., 2017a,b). Novel word meaning recall in incidental and even deliberate vocabulary learning is very low even after several encounters with the same word in a text (Lawson and Hogben, 1998; Webb, 2005, 2007; Elgort, 2011; Elgort and Warren, 2014). L2 speakers do not remember the meaning of these words well, apparently, because it is not robustly encoded, and a new LR with a fuzzy meaning resists consolidation and efficient storage in long-term memory (Qiao et al., 2009; Qiao and Forster, 2017). While L1 speakers, adults and children, also experience difficulties in novel word learning, L2 speakers deal with an additional set of difficulties. Thus, improper phonological encoding of LRs that we refer to as phonolexical encoding (in contrast with phonological encoding of individual word segments, see also, e.g., Llompart, 2021 for the use of this term) leads to nonnative patterns in the processing of similar-sounding words. More generally, improper phonolexical encoding influences the properties of nonnative lexical networks – irrespective of whether the LRs involve especially difficult L2 phonological contrasts (Pallier et al., 2001; Darcy et al., 2012; Mora and Darcy, 2013) or not (Gor and Cook, 2020). Given that the fuzziness in L2 lexical encoding of particular words may never be resolved for individual L2 speakers even when they reach high L2 proficiency levels, it is useful to incorporate the degree of fuzziness in lexical encoding as a property of numerous L2 words that interacts with the well-attested input frequency effects. Accounting for fuzziness in form and meaning encoding as well as in the mapping between them will contribute to a more efficient and accurate modeling of the learning trajectories for different types of lexical units, L2 learner profiles, and learning conditions, as well as explain the differences between L1 and L2 lexical processing [see below about the developmental trajectories for L2 LRs within the framework of the Ontogenetic Model (Bordag et al., 2021a,b)].

## Is the Construct of Flr New, or Does It Rename the Existing Constructs?

The FLR hypothesis builds upon existing research, primarily on L2 phonolexical encoding. The term *fuzzy lexical representations* has been previously used in the SLA literature, albeit in a limited sense – to refer to the specific phonological difficulties that L2 learners encounter when encoding problematic L2 phonological contrasts in LRs (Darcy et al., 2013; Llompart and Reinisch, 2019). The FLR hypothesis also draws on research that does not necessarily use the construct of fuzziness in phonological representations; however, it examines the consequences of nonnative phonological encoding, such as lexical confusions (Pallier et al., 2001; Escudero et al., 2008, 2014; Hayes-Harb and Masuda, 2008; Escudero and Wanrooij, 2010; Chrabaszcz and Gor, 2014, 2017). This strand of research explores the bottom-up direction in the encoding of LRs: L2 speakers are inefficient at the processing of L2 phonetic cues, which leads to problems with the phonological categorization of word segments and the identification of the phonemic sequences corresponding to the spoken word input. The phonological categorization problems, in turn, contribute to the poor lexical encoding of the words with difficult L2 phonemes or phonological contrasts (e.g., “rock” and “lock” are confusable for Japanese learners of English; Ota et al., 2009).

Importantly, the construct of FLRs has also been extended to refer to low-resolution L2 lexical representations that do not necessarily involve particularly problematic L2 segments, but nevertheless lead to lexical confusions of similar-sounding words (Cook et al., 2016). Such poorly encoded FLRs contribute to a nonnative pattern of lexical competition in phonological priming experiments (Gor et al., 2010; Cook and Gor, 2015; Gor and Cook, 2020).

The FLR hypothesis expands the construct of fuzziness to all levels of L2 lexical encoding: – phonological and orthographic form, as well as meaning – and also to form-meaning and phonological form-orthographic form mappings. Within this approach, fuzzy encoding interacts with input-based factors, such as lexical frequency or context predictability, to shape the pattern of L2 spoken word recognition and other aspects of L2 lexical processing. Note that the FLR hypothesis shares its focus on the quality of lexical encoding with the lexical quality hypothesis developed for L1 reading (Perfetti and Hart, 2002; Perfetti, 2007), and the lexical entrenchment hypothesis developed for written word recognition (Diependaele et al., 2013; Brysbaert et al., 2017). However, in contrast to the lexical entrenchment hypothesis, the FLR hypothesis treats the quality of lexical encoding as a product of several interacting factors rather than solely an outcome of input frequency.

The FLR hypothesis seeks to build a bridge between the acquisitional aspects of SLA research and word recognition studies. It posits that an L2-specific set of difficulties in lexical encoding and word recognition arises from two major factors shaping adult SLA: age of onset and L1 transfer.

### Late Age of Onset

The post-puberty age of onset of language acquisition is associated with lower learning outcomes for L2 (DeKeyser, 2012; Hartshorne et al., 2018; Bylund et al., 2021), with post-puberty learners failing to achieve native levels of proficiency on a battery of tests targeting different aspects of L2 linguistic knowledge, including L2 phonological sensitivity and control of idiomatic language (Abrahamsson and Hyltenstam, 2009; Bylund et al., 2021) and in lexical development (Bylund et al., 2019). Phonological acquisition is particularly vulnerable and shows early age effects (Granena and Long, 2013).

### L1 Transfer

The L1 mental lexicon is already in place when L2 lexical learning starts and so is the L1 phonological system. L2 learners need to overcome the influence of L1 in developing the L2 phonological system and new form-meaning mappings for L2 LRs. The specific difficulties in L2 lexical encoding of both form and meaning can often be traced to a particular combination of L1 and L2 (Jarvis, 2000; Barrios and Hayes-Harb, 2020, 2021; Llompart, 2021). For example, an L1 German speaker may not encode the difference in the English words *cod* and *cot* due to final consonant devoicing in German, while encoding this difference will not present a problem to an L1 French speaker. An L1 French speaker will be confused with the meaning of the English word *library*, because *la librairie* in French is a bookstore.

While a late age of onset and L1 transfer are not independent factors – L1 transfer is to be expected at an older age when L1 is already in place – they contribute to FLRs in different ways. A late age of onset is associated with reduced network plasticity in general and parasitic reliance on the L1 lexical network (Hall and Ecke, 2003), both potentially increasing fuzziness in lexical connections. In contrast, L1 transfer depends on a particular combination of L1 and L2 and manifests itself in issues with individual aspects of lexical encoding that have their source in L1 phonology, orthography, or semantics.

Another factor that mitigates novel word learning is L2 proficiency. On the one hand, L2 proficiency encompasses different kinds of linguistic knowledge, with lexical knowledge being part of it, since the level of L2 proficiency is associated with the size and the degree of familiarity of L2 vocabulary (Laufer, 1997; Alavi and Akbarian, 2012). On the other hand, vocabulary size by itself is also a predictor of subsequent lexical learning. As in “the rich get richer,” L2 learners with a larger vocabulary and a more elaborate lexical network are more efficient at the lexical encoding of novel words (Llompart, 2020; Daidone and Darcy, 2021). In this vein, the extent of fuzziness of individual LRs depends on their stage on each LR’s acquisition trajectory. The actual shape of the developmental curve for different aspects of the lexical representation depends on a number of factors, and these aspects do not necessarily develop in parallel. In section “How Can the Construct of FLRs Help to Improve the Existing Models of LRs and Lexical Access?,” we discuss how lexical encoding becomes more precise as L2 learners’ proficiency increases within the framework of the Ontogenetic Model (Bordag et al., 2021a,b).

The effect of age of acquisition (AoA) for lexical learning can be separated from the effect of lexical frequency or the cumulative number of encounters with the word (with the latter also depending on AoA). The role of AoA for L1 words was demonstrated in a megastudy using crowdsourcing technology that explored self-reported AoA for over 30,000 English words and showed that the AoA ratings explained a substantial percentage of the variance in the lexical decision data of the English Lexicon Project, over and above the effects of log frequency, word length, and similarity to other words (Kuperman et al., 2012). This effect of AoA in L1 above and beyond lexical frequency (and the cumulative number of encounters with the word) suggests that at an older age, lexical encoding and retrieval becomes less efficient. It is to be expected that the quality of lexical encoding will be less efficient across-the-board in adult L2 learners.

Fuzziness can be also viewed as a property that characterizes the L2 lexicon or a lexicon of a nonproficient L2 speaker in general. In this sense, the approach is related to the cognitive theories that address the differences in representation and processing in novices and experts. One such theory is the Fuzzy Trace Theory (FTT) by Brainerd and Reyna (2002). FTT is a dual-process theory that assumes two types of representation of past events: meaning-based gist representations, which support fuzzy (yet advanced) intuition, and superficial verbatim representations of information, which support precise analysis (Reyna, 2012, p. 332). Both types of representations are encoded in parallel, can be retrieved independently from each other, and have different forgetting rates (with verbatim traces becoming inaccessible at a faster rate than gist traces). It is important to note that despite the relevance of the theory to the topics discussed in this paper, the term “fuzzy” is used differently in the FTT. In the FTT, it relates to the processing of experts, who rely on a broad and deep knowledge foundation, from which they can derive the “gist” which the authors of FTT refer to as fuzzy. The novices, on the other hand, do not have such a rich knowledge base at their disposal and are thus more focused on the surface, form-based representations (cf. form prominence in L2 in section “Form Prominence in L2” and fuzziness decreasing over time in section “How Do FLRs Develop Over Time – Is Fuzziness Reduced?”).

The FLR hypothesis connects different strands of research on lexical encoding in L2 word recognition and vocabulary learning and frames the discussion of how to predict and measure fuzziness in lexical representations and incorporate it as an additional parameter in models of L2 lexical processing. The goal of the FLR approach is, on the one hand, to account for systematic patterns of fuzziness associated with specific encoding problems and, on the other, for random fuzziness also present in the LRs and lexical networks of adult L2 speakers. To summarize, the FLR hypothesis, while drawing on previous research, extends the construct of fuzziness to different aspects of lexical encoding of L2 words and, unlike other approaches, treats it as a property of L2 word encoding that interacts with other factors in vocabulary acquisition and processing.

## What Causes L2 Lrs To Be Fuzzy?

### Fuzziness in Form, Meaning, and Form-Meaning Mappings

When a learner encounters a new spoken word, the phonological form and the meaning of this word are encoded, and a connection between the form and the meaning is established. If the word is encountered only once and especially in noisy conditions – a property of naturalistic settings – its sound form may not get properly encoded. If the context in which the new word is encountered does not make it possible to unambiguously identify its meaning, it will also be encoded without proper specifications and details, maybe merely as a “place-holder” with broad semantic properties, such as a reference to a semantic field (e.g., “some kind of a gardening tool” and “a positive human character trait”). With more encounters with the word, its form- and meaning-related properties become better defined, and the encoding becomes more precise. In this respect, the word learning trajectory is similar in both L1 and L2 word learning.

At the same time, several factors specific to adult L2 learning contribute to increased fuzziness in L2 lexical representations. As we state above, these factors are globally defined by the late AoA of individual lexical items and the fact that L2 words are acquired when the L1 mental lexicon is already in place.

The existence of the L1 phonological system supported by a system of phonetic cues for the encoding of speech sounds as phonemes means that a lot of perceptual restructuring will be needed and new L2 phonological categorization routines will have to be established even for the L2 sounds that have correspondences in the L1 (Llompart and Reinisch, 2019; Llompart, 2020). According to the FLR hypothesis, two aspects of phonological processing in the L2 lead to less precise spoken word encoding: (i) problems with the phonological categorization of difficult L2 phonemes or contrasts (especially, in situations presenting an allophonic split problem, when two distinct L2 phonemes map onto a single L1 phoneme, i.e., in single-category assimilations – see Best and Tyler, 2007), and (ii) the overall imprecision of phonolexical encoding in L2 compared to L1 that may involve ambiguous word segments or their inexact sequence. Both aspects stem from the mismatches between the phonological systems of the L1 and the L2; however, the former has received more attention in the literature than the latter (see Llompart and Reinisch, 2019 regarding the role of phonetic flexibility in the robustness of L2 phonolexical encoding).

The first aspect manifests itself when adult L2 learners encounter a phonological contrast absent in their L1, such as the vowel /i/-/ɪ/ contrast in English that is absent in Spanish or French or the /y/-/u/ contrast in French that is absent in English. L2 learners’ perceptual systems are not attuned to processing the phonetic cues differentiating these phonemes, and the L2 phonemes may not be properly represented and contrasted in the L2 phonological system. This absence of phonetic attunement and/or robust phonological categories in L2 has two implications for lexical learning. First, phonological encoding of the words differentiated by this contrast is fuzzy, because the contrasting phonemes are not properly categorized (see Pallier et al., 2001; Sebastian-Gallés et al., 2006; Hayes-Harb and Masuda, 2008; Broersma and Cutler, 2011; Darcy et al., 2012, 2013; Sebastián-Gallés and Díaz, 2012). And second, given that phonological categorization difficulties persist over time, phonolexical representations of the words with problematic L2 contrasts remain fuzzy even at higher levels of proficiency (Chrabaszcz and Gor, 2014, 2017).

The second aspect has to do with a more diffused perceptual categorization deficit in L2 (resulting from the phonetic differences between L1 and L2 sounds and also language-specific phonotactics, segmentation, and lexical prosody), which leads to “summative,” less precise phonolexical encoding of novel spoken words (cf. coarse-grained orthographic representations that lack precise positional information, Grainger and Ziegler, 2011). This latter type of fuzziness in LRs is less systematic and more akin to white noise, as it makes the word encoding indistinct and leads to underdifferentiated LRs that are easily confusable not only with their phonological neighbors, but also with more distant similar-sounding words. The effects of such blurred phonolexical encoding in the absence of a particular difficult contrast leading to phonological confusion were reported in a study manipulating the Levenshtein distance (Levenshtein, 1966) between the matching Russian translation of an English word and its similar-sounding counterpart. It revealed that L1 Russian speakers and English-speaking L2 learners of Russian were differently sensitive to the “overall” phonological similarity between two L2 words. While Russian L1 speakers could be confused by two words with a Levenshtein difference of 1 (i.e., one phoneme substitution, addition, or deletion), L2 speakers were confused with the words with a Levenshtein difference of not only 1, but also 2 (Cook et al., 2016). Unlike the fuzziness in LRs resulting from particular resistant phonological difficulties, diffused spoken form fuzziness is reduced with more input. A similar pattern of lexical acquisition starting with low-resolution lexical representations that are improved during differentiation and sharpening was reported for L2 children (Baxter et al., 2021a,b). Meaning encoding in L2 is also characterized by L2-specific features. Thus, L1 speakers rely both on linguistic and on nonlinguistic context (i.e., on schemata, knowledge of the situation, or real-life knowledge) when they establish the meaning of novel words that they will encode in the LR. In contrast, lower-proficiency L2 speakers make inefficient use of the linguistic context because they do not understand it well and/or they fail to process it efficiently enough in real time. Multiple examples of the inability of L2 speakers to make use of the high close probability contexts to predict the upcoming word when the sentence is presented in noise is an illustration of the auditory processing constraints in L2, albeit in extreme conditions (see, e.g., Gor, 2014).

According to the FLR hypothesis, fuzziness in the encoding of form and/or meaning in L2 leads to fuzziness in form-meaning connections. As mentioned above, in L1, word forms and meanings are acquired together, which results in well-defined and strong form-meaning connections. While acquiring L1, children have to categorize both strings of sounds – to identify individual word forms – and portions of reality (objects, events, people, animals, etc.) that correspond to them; a child learning a new word, for example, “parrot,” simultaneously encounters the bird (possibly, its picture, or a toy), to which it refers. The form and the semantic components of the L1 lexicon thus develop simultaneously, resulting in amalgamated lexical entries with strongly connected semantic and phonological representations (Perfetti, 2007). In contrast, in L2, word forms and meanings are often acquired separately, with word meanings initially borrowed from L1 (Jiang, 2000). An adult L2 learner acquiring the new English word “parrot” may have its meaning already represented through experience with L1, in which case a new L2 word is mapped onto the already existing semantics. However, this is not a unique possibility. First, novel L2 words may refer to objects or concepts that do not occur in L1 and have no corresponding word in L1 – these are often culturally-specific words (e.g., *bar*/*bat mitzva*, “the Jewish coming of age ritual,” *Hanukkah* in Hebrew, *sutki* “24-h period” in Russian, *siesta* “an afternoon nap” in Spanish, and *bento* “a single-portion meal packed in a box” in Japanese). And second, the development of L2 semantic representations often starts by borrowing L1 semantic representations, which serve as a shortcut, although some restructuring and reconfiguration of the L2 semantic representations may be required at a later time, when more L2 input providing finer-grained information about the specific L2 meaning becomes available. For example, an L2 learner of English may first discover the loanword *kosher* in the general meaning of “legitimate” and later will discover that *kosher* has a very specific meaning referring to Jewish food that has been ritually prepared, or the discovery of the core and general-purpose meanings may occur in the inverse order. Complex relations often also exist between translation equivalents which are rarely completely equivalent in two languages. For example, an L1 speaker of Russian learning L2 English may initially map the concept of “a piece of furniture with a horizontal surface” to the L1 word *stol*, which is a semantic equivalent of the English *table*. However, with more experience with L2 English, the original mapping will have to be updated to reflect a difference in meaning for *table* vs. *desk* that Russian does not lexicalize.

**Figure 1:**
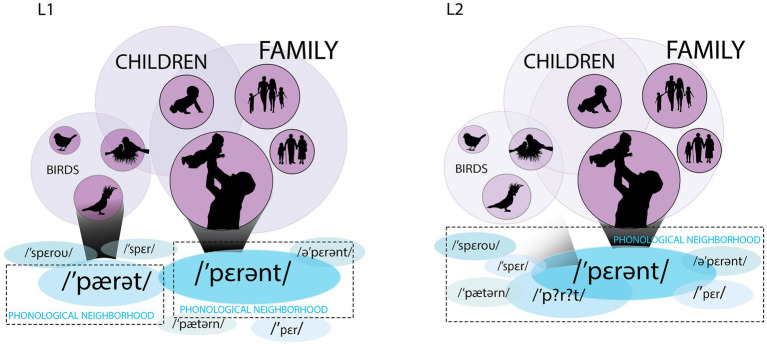
L1 and L2 lexical representations. Panel on the left represents the word *parrot* and a similar-sounding word *parent* in L1 (in British English), and panel on the right – the same words in L2. The blue ellipses at the bottom represent the phonological neighbors and similar-sounding words. The size of the ellipses represents the lexical frequency of the words. The mauve circles represent the semantic representations and their semantic fields, while the grey cones represent the activation spreading from the form to meaning. In L1, the words *parrot* and *parent* are differentiated at the phonological level and belong to different neighborhoods. Each word activates its corresponding meaning. In L2, both the form and meaning of the word *parrot* are fuzzy. It is phonologically encoded as /p?r?t/ and is likely to be confused with /pɛrənt/, a high-frequency and more familiar word. Semantically, /p?r?t/ can activate “parent” and “family,” but also “birds,” rather than “parrot” because the exact semantic referent is unavailable given the fuzzy semantic encoding.

Figure 1 is a schematic depiction of the lexical representation of the word “parrot” and a similar-sounding word “parent” in British English in the L1 and L2 lexicons. In British English, unlike many dialects of American English, these words are not phonological neighbors in strict terms, since their Levenshtein distance has a value of two (the difference in the /æ/-/ɛ/ vowels and the presence or absence of /n/, i.e., one substitution and one deletion/addition). In L1, the lexical representations are highly specified both at the phonological and semantic levels: Phonological form is encoded precisely allowing L1 speakers to efficiently constrain the word’s phonological neighborhood and activate it quickly. The words *parrot* and *parent* are disambiguated at the initial syllable due to the accurate encoding of the first vowel. The form /pærət/ has a strong connection to the semantic representation of “parrot.” The same is true for the word /pɛrənt/ that is strongly connected to “parent.” “Parrot” is associated with the semantic field of “birds,” while “parent” is associated with “family” and “children.” In L2, the phonological representations are fuzzy, and at the initial stages of acquisition, they can be characterized by imprecise phonological sequences involving the inclusion of incorrect or additional phonemes (or their exclusion) or a scrambled order of phonemes. At this stage, the word *parrot* is encoded as /p?r?t/, which means that two segments are fuzzy. As a consequence, the distinction between similar-sounding L2 words is also fuzzy, or blurred, and the more familiar word *parent* may be accessed instead of the intended less familiar word *parrot*. Similarity of forms leads to the blending of two phonological neighborhoods in L2 and the activation of similar-sounding words that would not be activated in L1 lexical access. In this example, L2 semantic encoding is also fuzzy, and accordingly, *parrot* is associated with the broader semantic field of “birds” rather than “parrot,” and moreover, if it erroneously accesses the semantics of “parent,” it may activate the semantic field of “family.”

The FLR hypothesis argues that the weak mapping links between L2 form and semantic representations are likely related to the specific starting conditions for their emergence in L2 acquisition. The focus of basic L2 word acquisition, especially at the initial stages, is on the word forms that need to be encoded, stored, and mapped onto the preexisting semantic representations borrowed from L1. Due to these developmental differences between L1 and L2 lexical acquisition, the mapping between phonological forms and semantic representations is weaker and fuzzier in L2. At the same time, since a substantial semantic store has typically already been developed during L1 acquisition, fuzziness at the semantic level does not have to be initially as pervasive in L2 as it was during L1 acquisition. When new L2 forms can be mapped to the existing semantic representations, fuzziness may arise only later when the L2 learner discovers that there is no complete translation equivalence between two given words and that an adjustment to the semantic representation needs to be made. Importantly, new semantic representations also emerge in L2 – both temporal (e.g., when only imprecise meaning can be inferred from the context, e.g., that a “parrot” is some kind of a bird, without the specific knowledge about the species) and longer-lasting (when new meanings are acquired through L2, for which there is no preexisting semantic representation; Bordag et al., 2018). Such fuzzy representations undergo stages in a similar way as do emerging semantic representations in child L1 acquisition. It is for further research to establish whether the form-meaning links differ in their strength depending on whether the form and the semantic representation were acquired successively or simultaneously.

The studies on lexical learning recur to semantic priming to gauge the robustness of semantic representations of newly acquired words (Elgort, 2011; Elgort and Warren, 2014; Bordag et al., 2015, 2017a). The absence of semantic priming or semantic inhibition is interpreted as evidence of poor integration of the newly encoded lexical representation into the semantic network, which is associated with its fuzzy semantic encoding. In fact, recent research (Elgort, 2011; Bordag et al., 2015, 2017a, 2018) has already provided evidence that different semantic priming effects in L2 emerge depending on several properties of the newly learned words associated with the quality of semantic encoding. In semantic priming tasks testing the integration of new L2 LRs into the L2 semantic network, the primes that were known existing words (Bordag et al., 2015), new primes, for which the participants could recall the meaning (2017a), and novel words with existing meanings (2018) produced facilitation in the processing of the targets, semantically related real words. By contrast, the primes, for which new semantic representations were established (2015, 2018) or for which the participants could recall only the orthographic form but not the meaning (2017a), produced inhibition (cf. also Carr and Dagenbach, 1990; Dagenbach et al., 1990a,b for L1). Therefore, better encoded, that is, less fuzzy new LRs show evidence of integration into the semantic network (facilitation), while the LRs with fuzzy semantic encoding slow down lexical retrieval of the target.

The discussion above has focused on the problems with the linguistic encoding of form and meaning and weak form-meaning mappings – all contributing to FLRs. The next section is devoted to a major factor shaping word learning – lexical frequency.

### FLRs, Lexical Frequency, and Lexical Entrenchment

Lexical frequency is estimated based on the frequency of word occurrence in a representative corpus. It is associated with word knowledge and the speed and accuracy of word recognition and retrieval from memory (see, e.g., Kuperman et al., 2012). Accordingly, one of the critical factors affecting L2 word learning, storage, and recognition is the reduced amount of L2 input leading to reduced subjective lexical frequencies (e.g., Ellis, 2002). A proposed explanation for the frequency effects evokes the notion of cognitive entrenchment – a cognitive consequence of increased exposure to a certain external stimulus. Every time a certain event occurs, its memory trace becomes more and more profound or entrenched. Since entrenchment is a function of repetition of cognitive events, units are variably entrenched depending on the frequency of their occurrence (Langacker, 1987; Tomasello, 2003). Higher levels of entrenchment are associated with a greater processing advantage. Conversely, a lack of entrenchment can lead to processing costs, which have varying implications for lexical access at different stages.

Cognitive entrenchment constitutes the core of the lexical entrenchment hypothesis (Diependaele et al., 2013; Brysbaert et al., 2017), which argues for the critical role of input frequency in determining the level of entrenchment of lexical entries in the L2 mental lexicon and focuses on the written modality. The lexical entrenchment hypothesis builds on the lexical quality hypothesis developed for reading in L1 (Perfetti and Hart, 2002; Perfetti, 2007) and treats lexical quality, or the quality of lexical encoding, as a direct product of the number of encounters with the word. It assumes that every new exposure to the word strengthens the form-meaning connections and contributes to stronger lexical entrenchment. Remarkably, Brysbaert et al. (2017) report that lexical information build-up is slower in L2 than in L1 and conclude that entrenchment in L2 may be qualitatively different from L1. This position, if further developed and substantiated by empirical evidence, may go in the direction of the acknowledgment of the increased role of fuzzy encoding in L2 compared to L1.

SLA research makes a distinction between input and intake in L2 (Corder, 1967; Gass, 1997), because for L2 learners, input processing in real time, and especially, processing auditory input, is effortful and error-prone. Depending on the L2 proficiency level, more or less auditory input is actually processed, that is, becomes intake. For spoken word recognition and learning, this means that only some L2 words in the input are noticed (Schmidt, 1990; Gass, 1997), understood, and lexically encoded. Accordingly, in contrast to the lexical entrenchment hypothesis and the computational models based on it, such as BIA+ and Multilink, the FLR hypothesis is built on the understanding that the quality of linguistic encoding in L2 is not determined solely by input frequency, but rather by a set of linguistic and cognitive factors, in addition to input frequency.

The factors that contribute to the processing of novel spoken words include the availability of the meaning for a new L2 lexical item and the relative ease or difficulty of phonological categorization and encoding given the combination of L1 and L2. According to the FLR hypothesis, the quality of lexical encoding interacts with input frequency, rather than automatically improves with more input. This interaction of the inherent difficulty of encoding, and in particular, phonological encoding of problematic L2 segments that is specific for different lexical entries with input frequency ultimately configures the properties of the LR in L2. Initial problems with encoding may be persistent, with some FLRs resisting lexical consolidation, in contrast to other LRs that are more amenable to robust encoding with sufficient input.

The quality of phonetic encoding has been shown to improve with increased input in young monolingual children (Swingley and Aslin, 2000; Garlock et al., 2001; White and Morgan, 2008) and adults (White et al., 2013). In a novel vocabulary learning experiment, monolingual English-speaking adults were trained and tested on nonword-nonobject picture pairings, with their eye movements monitored. Novel words were presented with various frequencies during training. In the testing phase, the participants showed no sensitivity to a one-feature phonetic mismatch in the presented test nonwords in their looking behavior. For higher frequency words, participants differentiated both one- and two-feature mispronunciations from correct pronunciations (White et al., 2013). This pattern can be partially extended to adult L2 learners; however, in L2 learners, input frequency does not solely determine the quality of phonological encoding of L2 words. As speakers of a particular L1, L2 learners experience difficulties in encoding particular phonemes and differentiating phonological contrasts, for example, *ship-sheep* is difficult to differentiate and properly encode for L1 Spanish speakers, and these difficulties persist even for high-frequency words. Thus, the quality of lexical encoding in L2 reflects the actual knowledge of the word, including its form and meaning, and depends on the word’s frequency in the input. Importantly, it is also a product of the word’s potential of being properly encoded given the particular combination of the L1 and L2, the linguistic properties of the word, such as the number of phonological neighbors, form salience, and imageability, the contexts in which it appears, and the proficiency level of the L2 learner.

## What Consequences Does Fuzziness in Lrs Have For Lexical Processing?

This section will address a range of issues observed in L2 word recognition and processing that the FLR hypothesis attributes to the fuzzy lexical encoding and form-meaning mappings. It will discuss the role of fuzziness in the nonnative patterns of lexical competition and lexical confusions, either transient or permanent. It will also connect fuzziness in LRs with the observations of form prominence in L2 and an increased reliance on recently engaged episodic representations in L2.

### Spoken Word Recognition in L2: The Effect of FLRs on Lexical Competition in L2

According to the FLR hypothesis, lexical activation, competition, and selection for L2 words are affected by fuzziness in form and/or meaning encoding that leads to weak or incorrect form-meaning mappings. Recall that the FLR hypothesis identifies different sources of fuzziness, with fuzzy form or meaning encoding leading to fuzzy form-meaning mappings and lexical confusions, and asynchronous acquisition of form and meaning leading to weak (but not necessarily incorrect) form-meaning mappings. First, FLRs are weak competitors; consequently, lexical competition in L2 spoken word recognition is weak despite the fact that irrelevant competitors may be activated by mistake (Gor and Cook, 2020). And second, as a consequence of weak lexical competition, L2 speakers over-rely on sublexical processing in resolving lexical competition. Below, we elaborate on these points.

The models of auditory speech perception agree that before the correct word is identified, several potential word candidates are considered (TRACE, McClelland and Elman, 1986; Cohort theory, Marslen-Wilson, 1987; NAM, Luce and Pisoni, 1998). The selected word candidate will have the highest activation level among the competitors. According to one point of view, L2 speakers show greater processing costs in accessing L2 words because they activate a larger number of words than L1 speakers, that is, they have to deal with larger competitor sets (e.g., Van Wijnendaele and Brysbaert, 2002; Duyck et al., 2008; Gollan et al., 2008, 2011; Schmidtke, 2014).

The fact that L2 speakers engage larger competitor sets in L2, which are presumably a source of increased lexical competition in L2, is typically attributed to a perceptual deficit associated with nonnative phonology, such as inaccurate representations of L2 phonemes (Pallier et al., 2001; Cutler and Otake, 2004; Cutler et al., 2006; Darcy et al., 2012; Díaz et al., 2012). For example, Broersma and Cutler (2008, 2011) proposed that as a consequence of reduced sensitivity to nonnative phonological contrasts, L2 competitor sets include words that typically would not compete for selection during L1 lexical access. The competitor set, therefore, is expanded by these “phantom” activations, for example, a near-word DAF activates “deaf” in “DAFfodil,” or words that arise at a word juncture (e.g., the near-word LEMP activates “lamp” in the phrase “eviL EMPire;” Broersma and Cutler, 2008, 2011). The L2 processing costs emerge from a greater competition due to spurious activation of irrelevant competitors. In contrast, Cook (2012) argued that phonological “foes,” that is, phonological neighbors that compete for selection and slow down lexical access in L1 may turn into “friends” in L2 because of their phonological underspecification, and speed up lexical access in L2.^3^

Several issues need to be considered with regard to a larger competitor set in L2 in the light of the FLR hypothesis. First, the FLR hypothesis also predicts that fuzziness in the encoding will lead to spurious activation of irrelevant competitors thereby potentially increasing the competitor set. However, it predicts weaker competition in L2 spoken word recognition. This is because due to the uncertainty associated with the fuzzy encoding and weak form-meaning mappings, the words in the competitor set will have low resting level of activation, and the activation of a larger set of words will be low and diffused. Indeed, L1 neighborhood research shows that high-frequency competitors are likely to negatively impact the speed of identification of a lower-frequency target, while low-frequency competitors produce no sizeable effect (Luce et al., 1990). Since L2 speakers are less exposed to L2 than L1 speakers are to L1, their L2 mental lexicon consists of the words that display characteristics of low-frequency words in the L1 lexicon (Gollan et al., 2005). Therefore, there are no reasons to expect that the competition between the L2 words will be stronger than in L1.

Second, the L2 lexicon^4^ of even high-proficiency speakers is generally smaller than the L1 lexicon, and consequently, the potential competitor set in L2 is more restricted than in L1. To illustrate the point, a well-educated native speaker knows about 20,000 word families, while highly educated nonnative speakers of English who are studying toward advanced degrees through the medium of English have a receptive English vocabulary size of around 8,000–9,000 word families (Nation, 2006). This comparison shows that the number of competing words in L2 should be considerably smaller than in L1, thus producing less competition overall. Thus, the effects of phantom activation of irrelevant words may be offset by a smaller competitor set in lower-proficiency L2 speakers.^5^

Third, the results of form priming experiments do not support strong lexical competition in L2. In phonological priming, onset overlap between the prime and the target leads to inhibition in L1, which is interpreted as an indication of strong lexical competition (e.g., Slowiaczek and Hamburger, 1992). Conversely, facilitation in form priming has been interpreted as a sign of weak lexical competition and an indication that sublexical facilitation dominates L2 processing of low-frequency words that are likely to have weak lexical representations in L2 (Gor and Cook, 2020; cf. Slowiaczek and Hamburger, 1992). For example, no significant inhibition was found in the group of L1 Dutch speakers for the L2 English minimal pairs, such as *flesh*-FLASH in cross-modal priming, although inhibition was observed in some individual participants (Broersma, 2012). In another study, L1 speakers of Russian consistently showed inhibition for phonological competitors with onset overlap in a phonological priming experiment, while L2 Russian speakers showed inhibition only for high-frequency word pairs, and facilitation for low-frequency word pairs (Gor and Cook, 2020).

To summarize, according to the FLR hypothesis, FLRs weaken lexical activation and competition in L2 lexical access and contribute to nonnative facilitation observed in phonological and orthographic priming tasks, with sublexical processes gaining more prominence in the L2. FLRs of newly acquired L2 words resist efficient consolidation and integration into lexical networks, which leads to the absence of the prime lexicality effect in L2 vocabulary training (Qiao and Forster, 2017) and to semantic inhibition when newly acquired lexical items in L2 with weak semantic representations serve as primes (Bordag et al., 2015, 2017a).

### Fuzzy Form-Meaning Mappings Lead to Lexical Confusions

We claim that lexical confusions, a well-attested phenomenon in L2 (Laufer, 1990; Laufer, 1997; Hemchua and Schmitt, 2006; Cook and Gor, 2015; Cook et al., 2016), happen when the form-meaning connections are fuzzy due to the fuzzy form encoding of LRs. Fuzzy form-meaning mappings fall into two main categories, each with its own consequences for lexical processing. First, the FLR hypothesis identifies fuzzy form-meaning mappings that are weak and lead to unstable connections between word forms and meaning. Often, the source of such weak connections is the fact that L2 word forms and meanings are acquired at different times (with the meaning initially borrowed from L1), and form encoding is not deeply entrenched (Diependaele et al., 2013; Brysbaert et al., 2017). Form-meaning mappings may also be weak for newly acquired LRs with insufficient number of exposures and/or insufficient consolidation period. When form encoding of many LRs in the L2 mental lexicon is fuzzy, or approximative, forms and meanings of similar-sounding words are not robustly connected, and as a consequence, lexical activation is weak. The selection of the LR from the list of activated candidates becomes more effortful, leading to longer RTs in word recognition and the reversal of the phonological priming effect, as in Gor and Cook (2020), and also error-prone, leading to transient lexical confusions that are difficult to repair. Second, the form-meaning mappings may be incorrect, which leads to permanent lexical confusions and mistakes in meaning recognition (Laufer, 1990, 1997; Cook and Gor, 2015). This section will focus on the findings documenting both transient and definitive lexical confusions in L2 lexical processing.

In addition to weak form-meaning mappings in L2 – when forms and meanings of L2 words are acquired at different times due to developmental reasons and are loosely connected – fuzzy encoding of phonological forms may contribute to a different aspect of FLRs: incorrect form-meaning mappings leading to lexical confusions. The transient lexical confusion effect was demonstrated in a pseudo-semantic auditory priming experiment by Cook et al. (2016). In this experiment, the prime-target pairs were semantically related through a virtual competitor that had a phonological onset overlap with the target, as in *korova* (“*cow*”)-*MOLOTOK* /malatok/ (“*hammer*”), with *molotok* “*hammer*” sharing its onset with *MOLOKO* /malako/ “*milk*.” While L1 speakers showed the same RTs for pseudo-semantic primes as for completely unrelated primes, L2 speakers showed a significant delay in RTs in the pseudo-semantic priming condition. The authors argued that L2 learners temporarily considered the pair *korova-moloko* instead of *korova-molotok* that was presented to them and had difficulty with abandoning this association when the input became incompatible with a semantically related onset competitor, likely because the phonological representations of the target word and/or its competitor were not sufficiently robust. Note that the visual world eye-tracking studies on English monolinguals that use a similar design to capture the transient activation of the semantic network of the phonological onset competitor point to phonological associations as the locus of the activation that engages the semantic network (Yee and Sedivy, 2006) (e.g., if the participants heard the word *logs*, they fixated on *key* because of the partial activation of *lock* absent from the visual display). In L2 word recognition, phonological forms of LRs can be associated based on the similarity of their sublexical features (see section “Spoken Word Recognition in L2: The Effect of FLRs on Lexical Competition in L2” above).

What is specific to L2 word recognition and what emerges from both eye-tracking and priming studies focusing on lexical competition is the pervasiveness of L2 speakers’ difficulty in resolving lexical competition. They do not efficiently and confidently identify the target word and abandon implausible competitors (Weber and Cutler, 2004; Cutler et al., 2006; Cook et al., 2016). According to the FLR hypothesis, the additional processing costs observed in L2 lexical processing are associated with the selection stage at which the LR is identified. Fuzzy encoding of form leads to fuzzy phonology-meaning mappings for individual LRs and impacts the functioning of FLRs in tasks involving word recognition. Thus, both visual world eye-tracking and pseudo-semantic priming experiments point to the same locus where transient lexical confusions that are difficult for L2 speakers to resolve originate – fuzzy phonological form encoding and fuzzy form-meaning mappings.

Further evidence for fuzzy mappings between word forms and meanings in L2 comes from experiments, which address the encoding of difficult phonological contrasts in orthographic representations of words, without involving any spoken input. While the main tenets of the FLR primarily concern spoken word encoding, fuzzy form-meaning mappings were also reported in experiments that involved no auditory input. A visual semantic-relatedness decision task (Ota et al., 2009) and a visual semantic categorization task (Ota et al., 2010) showed the effects of fuzzy phonological encoding which led to uncertainty regarding orthographic encoding of L2 words on the processing of L2 word meanings. The observation that incorrect semantic associations for English words, such as *key* and *rock,* emerged in the responses of L1 Japanese speakers who experience encoding problems with the /r/-/l/ contrast lends support to the idea that the LRs of *rock* and *lock* were fuzzy and not sufficiently separated in the mental lexicon. Crucially, while fuzzy phonological encoding is a result of perceptual categorization problems, auditory perception during the task completion could not be directly responsible for the semantic confusions reported by Ota et al. (2009, 2010). Accordingly, the results of the study speak in favor of FLRs being responsible for the confusions.

### Form Prominence in L2

This section will provide a quick review of the findings regarding form prominence in different populations of speakers that points to the same source – fuzziness in lexical encoding leading to weak form-meaning connections for less familiar words. Indeed, L2 speakers show a stronger preference for form-based associations for a number of reasons, all traceable to fuzzy encoding. The idea that the L2 lexicon is qualitatively different from the L1 lexicon was originally proposed by Meara (1978, 1983, 1984) based on a word association (WA) study with monolingual speakers and L2 learners of French. According to Meara, phonological links between words tend to play a much more prominent organizing role in the L2 mental lexicon than in the L1 mental lexicon. Several strands of research have reported since then that word form, whether spoken or written, has a greater prominence in L2 than in L1. Evidence in favor of form prominence in L2 mainly comes from WA studies (Jiang and Zhang, 2021); however, additional insights can be gained from form-based facilitation observed in morphological (Heyer and Clahsen, 2015; Li et al., 2017) and phonological priming experiments (Gor and Cook, 2020) and also from the comparison of memory for surface linguistic detail in L1 and L2 in longer texts (Bordag et al., 2021c). It should be noted that the majority of WA responses both by L1 and L2 speakers are still semantic in nature, indicating the importance of semantic networks both in L1 and L2 (Jiang and Zhang, 2021).

In WA studies, participants are typically asked to respond with one or more words to a given stimulus word. The type (and sometimes the number) of participants’ responses in WA tasks has been in the focus of L2 research for many decades (for an overview of WA in L2 research, see Fitzpatrick and Thwaites, 2020). Although the evidence accrued in this line of research, both with respect to L1 and L2, sometimes yields contradicting results, some patterns have been consistent. For instance, whether participants are more likely to respond to a given word either with a clang response (*mouse* – *mouth*), a syntagmatic response (*sit – chair*), or a paradigmatic response (*eagle* – *bird*) seems to be influenced by several factors. The probability of clang, or orthographic/phonological responses is increased in younger participants (Namei, 2004), if the cue word is relatively unfamiliar or newly acquired (Söderman, 1993; Wolter, 2001), or if the task is performed in L2 (Wolter, 2001; Fitzpatrick, 2006; Norrby and Håkansson, 2007; Jiang and Zhang, 2021). All these factors indicate that form-based responses are more likely in case of incomplete or unstable, that is, fuzzy representations. Syntagmatic or position-based responses are more likely to occur if participants respond in their L2 (Norrby and Håkansson, 2007; Zareva, 2007; Håkansson and Norrby, 2010) and have low L2 proficiency (Zareva and Wolter, 2012; Khazaeenezhad and Alibabaee, 2013). Paradigmatic, or meaning-based responses, including synonym responses, are more commonly observed for participants using their L1 (Fitzpatrick, 2006; Fitzpatrick and Izura, 2011) or in more proficient L2 speakers (Khazaeenezhad and Alibabaee, 2013), especially if they know the cue word well enough to use it in a sentence (Wolter, 2001), if they are expert users (L1 or advanced L2) of the language (Zareva, 2007; Jiang and Zhang, 2021), or if they are older [Namei, 2004, e.g., when they are adults as opposed to children (Cremer et al., 2011)].

Some of the mentioned factors are shared across L1 and L2, for instance, the observation that the better the word is known, or the older the speaker is, the more paradigmatic responses can be expected. However, some factors that change the proportion of responses are specific to L2: heritage L2 speakers (Kim, 2013) and more proficient speakers (Söderman, 1993; Zareva and Wolter, 2012; Khazaeenezhad and Alibabaee, 2013) are more likely to produce paradigmatic responses, while the proportion of syntagmatic responses is increased for speakers learning their L2 outside the target language environment (Håkansson and Norrby, 2010) or as a foreign rather than a second language (Norrby and Håkansson, 2007).

While semantic relations are at the core of the organization of the lexicon (as evidenced by the fact that semantic/paradigmatic/meaning-based responses are most prevalent across all studies, cf. Fitzpatrick and Thwaites, 2020), form- or syntagmatic (position-based) associations are more frequent, especially at lower acquisition stages (in children more than in adults), with less familiar words, and, importantly, in L2 compared to L1. Form prominence, as reported in the WA studies, can thus be related to fuzziness in LRs. Jiang and Zhang (2021) conclude that form is a more relevant factor in organizing the lexicon in L2 than in L1.

Additional support for form prominence and further evidence of the special status of form-based associations in the L2 lexicon comes from morphological priming studies that reveal reliable, purely form-based, orthographic priming effects in L2, while these effects are typically much weaker or missing in L1. For instance, Heyer and Clahsen (2015) observed facilitation in masked priming for purely form-related items (*career-CAR*) only in L2, while the facilitatory priming effects of the same size were found both in L1 and in L2 for morphologically and semantically related items (*darkness-dark*; for similar effects for compounding, see Li et al., 2017; however, for contrary findings see Diependaele et al., 2011). Form-based facilitation was also reported in phonological priming with onset overlap between the prime and the target (Gor and Cook, 2020).

Several factors can contribute to form prominence in L2. First, L2 learners can rely on the already existing L1 lexical system; therefore, when new L2 word forms need to be added, the already existing network of semantic representations can be engaged. Accordingly, the focus of acquisition is on the word forms that need to be stored and mapped onto the existing semantic representations. Since the semantic and the word form systems do not develop in parallel, they are less tightly connected in L2. Second, spurious activation of additional irrelevant competitors in L2 due to fuzzy phonolexical representations leads to more distributed and weaker activation of form-meaning connections. As a result, semantic representations that are activated through L2 word forms are activated less strongly than when the same representations are activated through L1 forms. Consequently, word meanings are less activated when processing L2, which foregrounds the form system and contributes to its prominence. Form prominence thus arises in L2 because of the reduced engagement of the semantic network compared to L1. The source of form prominence in L2 can also be traced to the specific role of episodic memory in L2 lexical processing, and the effort of L2 speakers to temporarily store rich detailed linguistic information because of their inefficiency in encoding and consolidating it for long-term storage. In the next section, we discuss how FLRs relate to different memory accounts.

### Fuzzy Lexical Representations, Episodic Memory, and the Complementary Learning Systems

FLRs are encoded and stored in memory, as are any LRs. However, fuzziness resists efficient memory consolidation and, therefore, may be responsible for the differences in how different memory systems subserve L1 and L2 lexicons. Several proposals underlying the differences in L1 and L2 lexical memory organization exist in the literature.

According to the episodic L2 hypothesis by Forster and colleagues (Jiang and Forster, 2001; Witzel and Forster, 2012), L2 words are represented in a different memory system than L1 words. In their studies, episodic recognition tasks elicit masked translation priming effects from L2 to L1 for “studied” L1 words but not for “unstudied” L1 words, whereas lexical decision tasks elicit asymmetrical effects in facilitatory priming from L1 to L2 but not from L2 to L1. Because L2 primes appear to be activated only in tasks requiring access to episodic memory, the authors conclude that all L2 words must be represented in the episodic memory system (or some other yet unspecified L2-specific memory system), whereas L1 words are stored in lexical memory. The effects, on which the episodic L2 hypothesis claims are based, turn out to be volatile: they have been reported for masked translation priming only under specific presentation conditions (Jiang and Forster, 2001; Witzel and Forster, 2012), but were absent in overt translation and semantic priming with two different SOAs and L1 Dutch-L2 English participants (Schoonbaert et al., 2009). Also, while no L2-L1 translation priming was observed for L1 Chinese speakers of English (Jiang and Forster, 2001; Witzel and Forster, 2012), an L2-L1 translation priming effect was observed for low-proficient L1 Korean learners of English (Lee et al., 2018). The limited evidence in support of the episodic L2 hypothesis seems insufficient to corroborate a major claim that L2 speakers rely on a different memory type in lexical processing compared to L1 speakers. More importantly, episodic memory is characterized by rapid decay over time and is only engaged in lexical processing within a short time span (Takashima et al., 2017) and, therefore, cannot replace long-term memory for the purpose of storing L2 LRs.

While episodic memory, by definition, cannot subserve long-term lexical storage, which challenges the viability of the episodic L2 hypothesis, a number of observations point to a greater reliance of L2 speakers on episodic memory in tasks engaging episodic representations of recently activated L2 words (Francis and Strobach, 2013; Bialystok et al., 2020). In recognition memory tasks, where participants have to recall recently studied stimulus words, L2 speakers perform better when recalling words in their L2, a less proficient language, and better than L1 speakers recalling words in their native language (Francis and Strobach, 2013). L2 speakers are also less likely to develop false memories of semantic lures (e.g., incorrectly recalling the word *needle* after studying the words *thread, pin, point*, and *sharp*) compared to monolinguals; however, they are more susceptible to phonological (form-based) memories (Bialystok et al., 2020), supporting the idea of L2 word form prominence discussed in Section “Form Prominence in L2”.

The FLR hypothesis maintains that certain properties of L2 lexical representations and the way they are acquired make them more likely to benefit from the demands imposed by episodic tasks. For example, form prominence (Jiang and Zhang, 2021) in the L2 lexicon (see discussion in Section “Form Prominence in L2” above) may help explain the priming asymmetry observed in the lexical decision tasks and the episodic memory tasks. In the episodic recognition tasks, participants have to identify the “old” words that they have studied vs. “new” words that they have not seen in the training set (but that they know). The task can be accomplished based on form recognition alone, without necessarily accessing the meaning. Thus, this task, where form recognition is critical, may be driven by form-based connections. Furthermore, greater episodic distinctiveness of low-frequency L2 words may result from a stronger novelty effect because frequency differences are subjectively greater in L2 (Francis and Strobach, 2013). The most likely reason why L2 speakers hold on to detailed episodic LRs is that they are inefficient at rapid and compact linguistic encoding of fuzzy LRs, and consolidation takes a longer time in L2. This last argument is supported by the Complementary Learning Systems (CLS) account (McClelland et al., 1995; Norman and O’Reilly, 2003) discussed below.

Evidence from word learning studies using consolidation paradigms suggests that there might be some differences between how L1 and L2 words are initially encoded in memory. According to the CLS account (McClelland et al., 1995; Norman and O’Reilly, 2003), memory traces are initially formed in the hippocampal and medio-temporal lobe (MTL) systems, which encode novel experiences (e.g., new words) immediately and support episodic memories. Over a consolidation period, these experiences are transformed into more stable representations supported by neocortical regions (temporal lobes). In L1 word learning studies, lexical competition exerted by newly learned words (e.g., *banara*) on the recognition of existing words (e.g., *banana*) after a period of consolidation results in inhibition, which is usually taken as evidence that a new word has been integrated into the mental lexicon (Gaskell and Dumay, 2003; Dumay and Gaskell, 2007; for a similar account, see Leach and Samuel, 2007). In L2, similar word learning paradigms yield different results. For example, Qiao and Forster (2017) observed that L2 speakers failed to show an inhibitory prime lexicality effect in a masked priming experiment in contrast to L1 learners (Qiao et al., 2009; Qiao and Forster, 2017). Instead, facilitatory (*banara* primed BANANA) and not inhibitory priming effects were observed in L2, suggesting that new words are not lexicalized or integrated into the lexical network in the same way in L2 as in L1, likely, because of less efficient encoding, that is, fuzziness.

Importantly, learning in the context of the CLS model depends on prior knowledge, or schemas – networks of interconnected, already existing neocortical representations that affect how new information is organized (Palma and Titone, 2020). The length of time during which new knowledge remains reliant on the MTL structures may depend on how well it fits a preexisting schema (Lindsay and Gaskell, 2010). Since L2 word learning is, by definition, subsequent to L1 word learning, encoding and integration of L2 words into existing memory may be mediated by the already existing schemas established during learning of the L1. Havas et al. (2018) explored this idea by examining the impact of existing phonological and semantic schemas on consolidation effects for words with familiar vs. unfamiliar semantics and with L1- vs. L2-like phonology. The authors found that both phonological and semantic aspects of word learning were enhanced by similarities with the existing schemas. For example, L1-like words were remembered better than L2-like words on the day of training (cf. the results of the study by McKean et al., 2013, in which children were also more accurate at a fast-mapping task for words with the phonotactics similar to their native language). These two findings suggest that the rate of initial encoding and later consolidation may differ for L1 vs. L2 word learning. Moreover, the relative engagement of episodic and semantic memory networks differs depending on whether only the word form or both the word form and its meaning were learned (Takashima et al., 2017). The fact that the reliance on episodic memory is increased for novel nonnative phonology and semantics provides support for the increased role of episodic LRs in L2 observed in the episodic memory tasks discussed above and for the association of episodic memory engagement and imprecise, or fuzzy lexical encoding.

## How Do Flrs Develop Over Time – Is Fuzziness Reduced?

The degree of fuzziness of LRs is related to their acquisition stage. Recently established and/or infrequently used representations are fuzzier than well-established, frequently used representations. The present article leaves out a detailed discussion of the developmental aspects of lexical representations due to space limitations. The developmental trajectories of individual LRs that depend on the linguistic properties of the LR and the learning context are captured by the Ontogenesis Model of the Lexical Representation (OM, Bordag et al., 2021a,b), and we refer the reader to these publications. The OM describes the ontogenesis of the LR within its phonological, orthographic and semantic domains, the mapping between them and with respect to their engagement in their corresponding networks. The OM assumes that most L2 LRs are fuzzy and that the ontogenetic curve of their development does not reach the *optimum* (i.e., the ultimate stage of their attainment with optimal encoding) in one or more dimensions. As has been discussed above, depending on the source of fuzziness, L2 LRs will be more or less amenable to more robust encoding with more input. The most “resistant” FLRs involve difficult L2 phonological contrasts or segments, which depends on a given L1-L2 combination. Such FLRs may continue to show poor phonolexical encoding even after extensive exposure to the spoken word. The OM focuses on unique developmental trajectories of individual LRs, hypothesizes that there is a developmental curve for each of the domains of a LR, phonological, orthographic and semantic, and proposes individual ontogenetic scenarios depending on linguistic and contextual factors [see, especially, Figure 5A,B in Bordag et al., (2021a)]. It extends the FLR hypothesis to the developmental domain.

The FTT, which is concerned with differences in the representation and processing of memories and decision-making in novices and experts (Brainerd and Reyna, 2002), takes a different approach to the role of L2 proficiency, or the acquisitional stage, in linguistic encoding that can be also applied to lexical encoding. The ability to derive the “gist” of a linguistic message seems to include several components: The ability to process the message and efficiently encode it verbatim, and then to extract the core meaning of the message, or the summary of its key points, and encode it as a compact “take-away” message. For spoken speech, the processing takes place in real time, thereby creating a high processing load that lower-proficiency L2 speakers cannot handle. It appears that the “universal” strategy of L2 speakers is to keep in memory a rich episodic representation that includes detailed, albeit imprecisely encoded form representations rather than to quickly package the semantic content of the received message. Note that low-precision phonological encoding with uncategorized raw phonetic details resisting consolidation is also characteristic of FLRs. Recent research on L2 text processing supports these assumptions. In a cued sentence recall procedure, Sampaio and Konopka (2013) showed that L1 and L2 speakers recall better the verbatim phrasing of sentences with nonpreferred lexical items (e.g., STRUCK vs. HIT) and are thus more sensitive to synonymous lexical substitutions in such sentences. Bordag et al. (2021c) directly compared memory for surface linguistic detail in L1 and L2 in longer texts and showed that L2 learners outperform L1 speakers not only in memory for lexical detail (cf. Sampaio and Konopka, 2013), but also for structural information. These findings indicate that L2 learners as novices are fixated on surface linguistic information (i.e., form-related), probably because they have to rely on reduced or inefficient access to the knowledge available in the semantic store.

## How Can the Construct of Flrs Help To Improve the Existing Models of Lrs and Lexical Access?

The FLR hypothesis proposes an extension to the existing models of L2 word recognition that are supported by network simulations, such as BIA+ and Multilink (Dijkstra and Van Heuven, 2002; Dijkstra et al., 2019) – an addition of the quality of encoding for different layers, as a parameter that interacts with input frequency rather than being its product. First, it should be noted that BIA+ and Multilink are developed for orthographic input, and thereby obviate one of the core issues in L2 LRs of spoken words – problems with phonological categorization. While the problem of proper orthographic encoding exists in languages with deep orthography, it is conceivably more amenable to training than phonological categorization that poses continuous problems for L2 learners, both for nonword segments (e.g., PAM-L2, Best and Tyler, 2007) and in phonolexical encoding (e.g., Darcy et al., 2012, 2013; Daidone and Darcy, 2021). Furthermore, these models represent an ideal L2 speaker, whereas in reality, even advanced L2 speakers may store inaccurate orthographic representations of words. Thus, neither BIA+ nor Multilink builds the quality of encoding into different levels of the lexical representation (different layers in the model) as an independent variable [e.g., contributing to the resting activation levels (Dijkstra and Van Heuven, 2002; Dijkstra et al., 2019)]. Rather, the quality of form encoding depends on the word frequency and its frequency ranking in the corpus (Dijkstra et al., 2019, p. 661). Multilink establishes the strength of the links between the levels of form and meaning in the lexical representation by taking into account the L2 proficiency level under the same assumption that L2 proficiency is associated with exposure to L2, that is, with input frequency.^6^ The majority of the existing computational models of L2 word recognition are not concerned with modeling the quality of lexical representations in a developing L2 lexicon within the L1 neural environment depending on AoA. In contrast, Zhao and Li (2007, 2010) have implemented three variants of a self-organizing neural network model: with simultaneous, delayed, or late AoA of the L2. Their main finding was that when the AoA was early, then functionally distinct lexical representations could be established for both languages; however, if the AoA was late, the model was unable to recruit sufficient resources to entirely remap the existing L1 lexical network. L2 phonological representations were forced into the spaces unoccupied by L1, where accurate access and retrieval was made difficult, and chances of confusion were high because of how densely the conceptual space was populated – LRs were imprecise, and the boundaries between them were fuzzy. These findings are in agreement with the FLRs hypothesis, as it focuses on adult (i.e., late) L2 lexical acquisition and processing. Furthermore, the FLR hypothesis proposes a complex set of assumptions – the inherent variability, ambiguity, and imprecision of LRs in L2, that is, their fuzziness should be accounted for not only in the connection weights representing the qualitative aspects of mappings, but also in the direction and the quantity of these mappings, as well as their dynamic nature (see also Duta and Plunkett, 2021 about building a dynamic mapping between phonological and semantic representations in a bottom-up fashion into a neural model of spoken word recognition). The FLR hypothesis argues that form-meaning mappings in L2 are subserved by diffused activation engaging a greater number of form-level nodes due to larger competitor sets, as well as nonnative-like patterns of activation drawing on fuzzy phonolexical encoding. One possibility to build fuzziness in L2 phonolexical representations into a computational model of L2 spoken word recognition is to add the intake layer (see section “FLRs, Lexical Frequency, and Lexical Entrenchment”), where words will have ambiguous or incorrect encoding specific for a particular L1-L2 combination, to the model above the input layer (cf. Bordag et al., 2021a,b). The assumptions of the FLRs hypothesis, and specifically, the role of encoding at different levels of the LR and the consequences of fuzzy encoding for establishing form-meaning connections, and more broadly, for word storage and retrieval could be used to further develop the existing models of bilingual LRs and word recognition and to model L2-specific features of LRs, such as fuzziness, or imprecise encoding and mappings between the levels of LRs.

## Final Remarks

We have reviewed a number of phenomena reported in L2 word processing that point to the same origin – problems with lexical encoding in L2. The quality of encoding, the core property of lexical representations, according to the FLRs hypothesis, has also been evoked in several influential approaches to written word recognition, such as the lexical entrenchment hypothesis (Diependaele et al., 2013; Brysbaert et al., 2017) and the lexical quality hypothesis (Perfetti and Hart, 2002; Perfetti, 2007). While both the FLR hypothesis and the lexical entrenchment hypothesis acknowledge the role of input frequency in the quality of lexical encoding, they diverge in that the FLRs hypothesis argues for a certain independence of the quality of lexical encoding from word frequency. Indeed, it is true that L2 speakers are exposed to reduced L2 input, a major source of lexical fuzziness, and it also comes at a later age, and with the lexical system of L1 already in place – for all these reasons, L2 LRs are expected to be imprecisely encoded, or fuzzy. At the same time, according to the FLRs hypothesis, whose primary focus is spoken word storage and retrieval, if the source of fuzziness is a problematic phoneme or phonological contrast that entails difficulties in L2 perception (and typically, production as well), increased exposure to the word may not improve the quality of its phonolexical encoding or will improve it at a much slower rate. Given that weak phonolexical encoding leads to fuzzy form-meaning mappings, it is to be expected that the LRs of such words will remain fuzzy with increased input frequency. Meaning encoding may also develop slower in L2 because of the complex relations between the senses of L2 words and their L1 counterparts. The existing L1 semantic mappings may resist remapping as a result of decline in brain plasticity or because lower-proficiency L2 speakers fail to process spoken input efficiently and to take advantage of the context to build complex semantic representations of L2 words.

There is an important caveat to the claims that FLRs are observed, or even observable, in spoken word recognition tasks where deficits in online perception can be responsible for the outcomes ascribed to the stored LRs. While it is indeed impossible to determine whether the tasks using spoken words as input show the effects of lexical encoding of stored LRs, or online processing difficulties, or both, several data sets point to the unique contribution of the properties of stored LRs to the observed effects. These data sets, discussed above, rely on cross-modal priming that is argued to engage central representations rather than access representations (e.g., Broersma, 2012) and on semantic relatedness and categorization tasks that use visually presented words with underlying confusable phonological contrasts (Ota et al., 2009, 2010).

To summarize, the FLR hypothesis maintains, the quality of lexical encoding is the core property of L2 LRs that deserves further study. For example, initial problems with difficult L2 phonological contrasts leading to fuzzy L2 phonolexical encoding may persist over time, and phonolexical encoding is not improved with additional input. While such phonolexical encoding problems have obvious consequences – weak or incorrect form-meaning mappings in the L2 mental lexicon – they also impact all aspects of lexical retrieval: lexical activation, competition, and selection. Crucially, the encoding at all levels of FLRs may undergo later remapping resulting in new sources of fuzziness, as in semantic reconfiguration when new meanings are added. By using this approach, in which the quality of lexical encoding is not a direct product of more encounters with the word, but rather a combination of the linguistically driven encoding difficulty with input frequency, the models of LR and lexical processing will make it possible to explore how the quality of encoding and input frequency interact for different lexical units in L2.

No one set of behavioral evidence can fully test the FLR hypothesis given that it is making inferences about lexical representations that are not directly open to observation based on the processing data. Several lines of research and kinds of evidence need to be considered, which calls for a comprehensive program rather than a single test. Many studies reviewed in the manuscript test the FLR hypothesis; however, it is only by looking at the pattern of findings across several studies that we can claim that the FLR hypothesis receives empirical support. There are several kinds of evidence in support of FLRs identified so far:

- Lexical confusions in auditory word recognition (Darcy et al., 2012, 2013; Cook and Gor, 2015);
- Accuracy and speed in word recognition that suggest nonnative patterns in lexical activation, competition, and selection:
- A reversal of the phonological priming effect for less frequent/less familiar words from inhibition to facilitation interpreted as evidence of weak lexical competition and strong reliance on sublexical processing (Gor and Cook, 2020);
- A reversal of the semantic priming effect from facilitation to inhibition in semantic priming for newly learned words (Bordag et al., 2015, 2017a) and in pseudo-semantic priming for less frequent/familiar words (Cook et al., 2016);
- Semantic confusions in visual word processing in semantic relatedness and categorization tasks (Ota et al., 2009, 2010);
- Lexical confusions and overreliance on the sentence context to accept context-mismatching phonological neighbors of the target words with fuzzy phonolexical representations (Chrabaszcz and Gor, 2014, 2017).

These observed effects point to two main sources of fuzziness in L2 LRs: phonological encoding problems and semantic encoding problems. The main challenge in testing the FLR hypothesis is to tease apart the loci of fuzziness – the representational level or the perceptual level – which are confounded in experiments that rely entirely on auditory input. One way to differentiate the component of online perceptual difficulties from the representational deficits is to use orthographic input instead. This has been done in semantic relatedness and categorization tasks that engaged visual word processing to show semantic confusions (Ota et al., 2009, 2010) and in a visual semantic priming task (Bordag et al., 2015, 2017a). Another possibility would be to use visual primes in a cross-modal or visual masked priming experiment rather than auditory primes. The use of orthographic input would be justified for highly controlled orthographic stimuli to avoid potential orthographic encoding difficulties.

New and more focused research will provide additional behavioral and neurolinguistic evidence supporting fuzziness at the encoding, that is, the representational level in addition to the perceptual/processing level. Lexical confusions associated with orthographic encoding problems and the phonology/orthography interface also need to be tested. An ERP study of N400 effects for phonologically confusable incongruent lexical substitutions and the role of different factors contributing to the lack of sensitivity of L2 speakers to such substitutions will test the role of FLRs in sentence processing. Additionally, different dimensions of fuzziness of LRs can be further explored by comparing the performance of multiple native language groups on multiple phonological contrasts (similar to the approach of Barrios and Hayes-Harb, 2021). Word training studies can manipulate the hypothesized degree of fuzziness for L2 words (e.g., based on phonological contrasts or L1 transfer predictions) to examine how FLRs change over time and what consequences fuzziness has for their long-term maintenance in memory.

In the future, we plan to broaden the claim regarding fuzzy L2 lexical representations to potentially involve less explored nonlinguistic extensions of lexical representations that are processed and encoded in the sensory-motor and emotional systems and rely on different sensory pathways. Sensory pathways and emotions appear to be coactivated in parallel with the lexical representation in L1 (Altmann et al., 2012; Kuperman et al., 2014) and to a significantly weaker degree, in L2 (Sulpizio et al., 2019; see also Conrad et al., 2011; see however, Ponari et al., 2015).

In Section “How Do FLRs Develop Over Time – Is Fuzziness Reduced?,” we argued that L2 lexical representations and lexical processing seem to be more oriented toward the surface, form level and we related this observation to a more general difference between novices and experts, as described in cognitive theories, such as FTT. We maintained that this orientation could be due to the fact that L2 learners cannot access the information that is stored at the semantic level to the same extent as L1 speakers. In addition, studies on emotions and L2 report greater emotional and cognitive distance in L2 compared to L1 (e.g., Harris et al., 2003; Puntoni et al., 2009; Caldwell-Harris, 2014; Hadjichristidis et al., 2015; Hayakawa et al., 2017). These findings lend themselves to various interpretations. First, this increased emotional and cognitive distance in L2 could be a consequence of typically different acquisition contexts, in which L1 and L2 are acquired: L1 is acquired in emotionally varied and rich contexts, while L2 is often acquired in a more emotionally neutral classroom environment (Ivaz et al., 2016; Dylman and Bjärtå, 2018). Another, not mutually exclusive explanation is based on the claim that L2 processing is more taxing on cognitive resources compared to L1 processing (see, e.g., Morishima, 2013), which results in limited resources available for the processing of emotions (see Yates et al., 2010).

Rather than explaining emotional distance through the cognitive load and resource allocation in L2, the FLR hypothesis suggests that due to the fuzziness at the form level that results in diffused spreading of activation among not closely related word forms and weak form-meaning mappings, less activation reaches the semantic network. As a consequence, the sensory-motor features associated with the word semantics do not become sufficiently activated in L2. It is likely that emotionally relevant representations can be activated both within the lexical-semantic system (e.g., *darkness* – *fear*/*danger*) and nonlexical, sensory-motor, and emotional systems. It is a question for future research to explore the hypothesis that L2 lexical representations are more emotionally “flat,” because they are only weakly connected to the sensory-motor and emotional systems and/or because less activation is available in the semantic and sensory-motor systems to reach the corresponding features due to the fuzziness effects on the form and form-meaning mapping levels.

## Data Availability Statement

No original data were reported in the article; further inquiries can be directed to the corresponding author.

## Author Contributions

All authors contributed to the article and approved the submitted version. All authors conceived the original idea and contributed with drafting separate subsections of the paper, editing and proofreading. KG developed the theory and took the lead in writing the manuscript. KG coordinated and directed the project. SC created supporting visual content. DB provided financial support.

## Funding

This article was funded by the Publication Fund of the University of Leipzig. The work on this manuscript by KG was supported by funding from the Slavic and East European Language Resource Center (SEELRC) at Duke University. The contribution of Anna Chrabaszcz was funded by the Center for Language and Brain NRU Higher School of Economics, RF Government Grant, ag. No. 14.641.31.0004.

## Conflict of Interest

The authors declare that the research was conducted in the absence of any commercial or financial relationships that could be construed as a potential conflict of interest.

## Publisher’s Note

All claims expressed in this article are solely those of the authors and do not necessarily represent those of their affiliated organizations, or those of the publisher, the editors and the reviewers. Any product that may be evaluated in this article, or claim that may be made by its manufacturer, is not guaranteed or endorsed by the publisher.

## Footnotes

^1^We are considering mostly spoken word encoding and processing, thereby narrowing the scope of the discussion to hearing people.

^2^Sensitivity to the morphological structure of morphologically complex words is another potential source of fuzziness in the lexical encoding. This is a major and controversial aspect of word structure, and it will remain outside the scope of this publication.

^3^Phonological neighbors with underspecification of phonological form in L2 may include homophones that are not differentiated in form, while they have distinct meanings (see Pallier et al., 2001). At the same time, most studies exploring phonological underspecification leading to lexical underspecification and to lexical confusions show an asymmetry associated with the degree of phonetic overlap between the L2 and L1 phonological units. In the case of *sheep-ship* in L1 Spanish speakers, *sheep* will be selected over *ship*, but not the other way round (cf. Barrios et al., 2016), suggesting that the two words are not completely homophonous.

^4^This article does not address the issue of whether there are two separate lexicons corresponding to L1 and L2 or one lexicon encompassing both languages; at the same time, it supports the strong connection between the two lexicons.

^5^In lower-proficiency L2 speakers, L1 words may also be activated in the auditory modality. It is less obvious how strong the activation of L1 words is in highly proficient L2 speakers given that phonetic encoding serves as a strong cue, making it possible to tag the word as belonging to a particular language. With this in mind, the FLR hypothesis focuses on the L2 mental lexicon in the hope that more conclusive research on how phonetic tagging works (or does not work) in auditory perception will clarify the role of the L1 mental lexicon in L2 spoken word recognition.

^6^Also, while these models focus on the interactions between the L2 and L1 lexicons and lexical representations, the FLR hypothesis is mainly concerned with L2, even though it assumes that L2 and L1 mental lexicons are interdependent given that the L2 lexicon develops when the L1 lexicon is already in place. Accordingly, the mapping issues between the L1 and L2 lexical representations are relevant for the structure of L2 LRs because the L1 and L2 lexicons coexist in one mental space and interact.
